# Medical Opinion and Movement

**Published:** 1909-07-17

**Authors:** 


### Medical Opinion and Movement.

In an interesting contribution to the *American Journal of Surgery*, Dr. J. M. Barnhill, of Ohio, discusses Ochsner's Treatment of Appendicitis. He appears to be justified in his supposition that Ochsner's methods of treatment are not properly understood by a certain proportion of the profession, and his authority and records have been referred to in support of non-operative treatment of appendicitis. One of the essential principles of Ochsner's teaching is to operate early; in fact, he adopts the extreme surgical standpoint, that operation should be performed as soon as a diagnosis of acute appendicitis has been made. But he differs from the current text-book teaching in regard to cases which come under treatment after the infection has extended beyond the tissues of the appendix, and especially in the presence of beginning diffuse peritonitis. In such cases he advocates delay in surgical intervention until the patient's condition has been sufficiently improved to render operation safe. He does this because he claims to have medical measures at his command which are able to effect such an improvement and justify delay—namely absolute fasting by the mouth, and rectal feeding and rectal saline injections. In cases of severe vomiting he resorts also to gastric lavage. In all cases of appendicitis he deprecates the administration of food or cathartics by the mouth as long as there is any pain or other evidence of inflammation. Rest being one of the first essentials of treatment for any form of inflammation, everything should be done to ensure absolute rest of the bowel, and so prevent spread of infection. Briefly, therefore, the chief features of Ochsner's treatment are to withhold everything by the mouth and administer food and saline by the rectum, operate in all ordinary acute cases; but when there is evidence of spread of infection to the general peritoneum wait for a subsidence of severe symptoms by the measures above mentioned.

---

The use of Ventilators with revolving fans has become very common in large public buildings, especially restaurants and shops. In most cases these ventilators communicate with the outside, and they are installed, of course, with the idea of ventilating and purifying the air within by increasing the rate of exchange with the external atmosphere. But revolving fans are also frequently used, especially in hot weather, simply to produce a cooling effect by setting up a current of air inside. Drs. A. Sartory and A. Filassier have examined the bacteriological effect of these fans and ventilators upon the atmosphere within, and have communicated their results to a recent meeting of the Société de Biologic. They find that these appliances enormously increase the bacterial content of the air. Their experiments were numerous and varied, but it will suffice to refer to one or two. Thus in a restaurant of 400 cubic metres analysis of the air before the fan was working showed 12,500 bacteria per cubic metre; after working the fan for one hour 23,000 bacteria, an[illegible] after two hours 45,000 bacteria. In another café [illegible] 600 cubic metres the number of bacteria rose fro[illegible] 12,000 to 39,000 after the fan was working f[illegible] one hour. In every instance, in fact, the numbe[illegible] of bacteria per cubic metre was doubled, or eve[illegible] quadrupled. As the authors point out, with thes[illegible] facts such appliances cannot but be regarded a[illegible] dangerous and detrimental to the public health.

---

The favourable results obtained by Electrotherapy according to the method of Apostoli, in the treatment of hæmorrhage from uterine fibroids have le[illegible] Dr. P. Galante, of Naples, to try the hæmostati[illegible] effects of the electric current in cases of hæmoptysis He uses a constant current from thirty elements wit[illegible] a galvanometer. A large electrode formed of a dis[illegible] of zinc, covered with several layers of gauze an[illegible] saturated with distilled water, is placed on that par[illegible] of the chest which physical signs indicate to be th[illegible] seat of the trouble, and is connected with the positiv[illegible] pole of the battery. The negative electrode is placed on the back or some other convenient part of th[illegible] body. The strength of the current should be 15 t[illegible] 20 ampères, or, if the electrodes are sufficiently large as much as 50 ampères may be used. The duratio[illegible] of the application was 10 to 20 minutes. Dr. Galante reports details of two cases successfully treated i[illegible] this way. The first was that of a young man with a considerable infiltration at the right apex. He had had repeated attacks of hæmoptysis, and finally a persistent hæmorrhage set in, which all ordinary means failed to stop. On the fifth day galvanisation of the chest was carried out in the manner described, and the effect was immediate. The hæmorrhage ceased, and the cough was also markedly relieved. The following day there was again a tendency to hæmoptysis, which yielded at once to a second application. The electric treatment was repeated for two or three days, and there was no return of the hæmorrhage. The second patient, also a man, showed dulness and diminished respiratory murmur over the left lung. For some years the patient suffered from persistent hæmorrhages, which often lasted a month or more in spite of treatment. Application of the current immediately arrested the hæmorrhage and relieved the cough.

---

Attention has recently been called by Carter Rowley to an early sign of Tabes Dorsalis first described by Abadie in 1905, which consists in analgesia of the tendons, and of the tendo Achillis in particular, to pressure. The author finds that absence of sensibility in the tendo Achillis is found in two-thirds of all cases, a proportion which is similar to that of the loss of knee and Achilles' jerks, and the presence of Argyll-Robertson pupils. Analgesia of the biceps tendon is less frequent, but a diminished sensibility can be frequently made out. Moreover, examination of over 100 normal individuals shows that this bicipital analgesia is frequently present in the healthy. The earlier results of Negro and Racine show that diminished sensibility of the tendo Achillis occurs respectively in three out of ten, and in 17 out of 33 cases of tabes dorsalis, so that this phenomenon of Abadie can be considered as an early sign of the disease, and should be looked for as carefully as the other signs, such as loss of knee and Achilles' jerks, Argyll-Robertson pupils, and lightning pains.

---

A NEW Method for attempting to secure Sphincteric Control after Colostomy is described in the *Lancet* by Mr. Charles Ryall. The rectus abdominis is split vertically, and the sigmoid is drawn out and divided at a convenient point. The author believes that much of the difficulty in the management of a colostomy opening is due to the bulky spur produced by the older methods, and favours the division of the bowel and the closure of its lower end, which is then returned to the abdomen. The upper segment is then made less bulky by removal of appendices epiploicæ and mesenteric fat; but without in any way interfering with the blood supply. A loop of muscle fibres is then separated from the posterior aspect of the rectus on each side of the wound, and the centre of each strand is drawn across to the opposite side of the wound so that one loop overlaps the other. Through the ring thus formed by the overlapping muscle strands the bowel is passed, and the whole is sutured so as to preserve this disposition. Anchoring stitches are inserted through the skin and muscle inside to keep the bowel in position. The wound is then closed above and below the bowel, and the cut edges of the latter are sutured to the skin. The author claims that by this device a double sphincter is formed, consisting of both longitudinal and circular fibres. The longitudinal fibres are those of the anterior portion of the rectus, and the circular fibres are those of the loops from the posterior portions. A similar operation can be, and has been, carried out through the external oblique; and analogous proceedings are of course quite feasible for gastrostomy and appendicostomy.

---

THE Parotitis which is especially common in patients suffering from gastric ulcer is dealt with by Drs. H. D. Rolleston and Oliver in the *British Medical Journal*. From an analysis of the records of 1,000 consecutive cases of gastric ulcer treated in St. George's Hospital, they find that whereas in 470 patients treated by rigid oral starvation the percentage of secondary parotitis was 4.5, in 530 allowed something by the mouth—albeit water only in most instances—the percentage of this complication works out at 0.4. Moreover, only about two-thirds of the patients who develop parotitis have suffered actual hæmatemesis, which is therefore held to be of less importance in the ætiology than rigid starvation. Attention is drawn particularly to this because it has been supposed that hæmatemesis disposes to parotitis owing to post-hæmorrhagic leucocytosis and consequent thrombosis. The dry condition of the mouth is thought to be the principal cause of parotitis. This conception does not, however, explain the incidence of parotitis after certain laparotomies, notably ovariotomy and other pelvic operations; for oral starvation can hardly explain these also. The authors conclude that mouth washes do not prevent the occurrence of secondary parotitis. Another of their observations is that the condition is more often unilateral than bilateral. Both these facts would seem to require some explanation which the "dry mouth" hypothesis does not provide.

---

FOR a long time it has been known that Epilepsy, usually of the Jacksonian type, may occur in the tertiary stage of syphilis. A second and less common type of epilepsy occurring in the secondary stage of the disease also exists, and in the *Gazette des Hôpitaux* Guénot gives an interesting description of such a case. This form of epilepsy closely resembles the essential type except for the absence of the initial cry and aura. Symptoms of "petit-mal" such as vertigo and absent-mindedness, are also lacking. The fits may occur within three weeks of the appearance of the chancre, and often precede the usual secondary symptoms. The number of attacks is very variable, and to a large extent depends on the precocity and intensity of mercurial treatment. One of the best diagnostic points lies in this ready response to treatment, for the fits are definitely curable by mercury and no after effects are left. Prognosis is therefore good, which is in marked contrast to fits as tertiary symptoms, in which it is always grave. The condition is considered by Fournier, who first described it, to be a neurosis, a false epilepsy comparable to other neuroses which are attributable to syphilis. The cerebro-spinal fluid shows no trace of the treponema pallidum, but a slight lymphocytosis such as is found in all cases of syphilis is seen. More important evidence can, however, be obtained by Wasserman's hæmolytic reaction, for, although this reaction is usually positive in the secondary stages of the disease, the author obtained a negative result before the disappearance of the fits. A second test performed when these had ceased proved positive. It is, of course, unfair to generalise from the observation of a single case, but the results obtained by this reaction are sufficiently important to justify further research into the syphilitic nature of epilepsy, and, by repeated examinations at sufficient intervals of time, to follow the course of the affection and control its cure.

---

CYCLICAL Vomiting in Childhood is an obscure condition of which the published cases have been mounting up of recent years, and the pathology and ætiology of it seem to be still matters of very considerable doubt. Dr. Eleanor Jones reports such a case in the *Archives of Pediatrics* with an account of the findings at autopsy: fortunately not very many of the cases end fatally, and for that reason every one that is followed out completely is, in the present state of our knowledge, worthy of publication. The child, aged three, was in good health until after partaking of some mutton stew, when he began to vomit at irregular intervals for four days. Then he developed twitching of the extremities and head retraction: combined with obstinate constipation the syndrome suggested tuberculous meningitis, but presently the symptoms, with the exception of vomiting, passed off, and the diagnosis of toxic vomiting was made. Consciousness was not lost, nor was there any complaint of headache; and the knee-jerks were normal. The temperature ranged between 97° and 101° F. The spleen was not palpable, and the abdomen was soft, flat, and retracted. Urine was secreted scantily, and voided involuntarily: it contained no albumin, casts, or acetone. On post-mortem exmination almost the only abnormality found was advanced fatty degeneration of the liver, the structure of which under the microscope was almost indistinguishable. In view of the parallel which has been drawn by some authors between this condition of cyclic vomiting and delayed chloroform poisoning, it is interesting to note that in the former as well as in the latter the presence of acetone in the urine is not invariable. Probably both conditions are due to hepatic insufficiency or degeneration; but whether cyclic vomiting is the expression of a derangement of the liver which in those less liable—but still not normally resistant—can be evoked by the toxic effects of chloroform is a question which must remain as yet unsettled.

---

The Surgical Treatment of Tuberculosis of the Epididymis and Testicle is considered at length by Dr. C. G. Cumston in the *Annals of Surgery*. This writer formerly upheld the practice of early castration in cases of unilateral infection, in accordance with the view widely held that by this means dissemination of the bacilli to the prostate, seminal vesicles, and the other testicle may be prevented. For the last five years, however, he has abandoned this line of treatment, and is well satisfied with the results which he has obtained by epididymectomy when the testis itself is not involved in the tuberculous process. This latter fact is ascertained by splitting open the organ, and suturing it should it turn out not to be affected. This preliminary exploration of the testis is essential before the epididymectomy is attempted. The author lays stress on the fact that sexual power is not lost in those whose epididymis is affected by tuberculosis, although it may be in gonorrhœal lesions in this situation. He says that recurrence of the disease is no more frequent than when castration is done, and that the influence on the general condition and on the prostate or seminal vesicle if diseased is quite as good. He emphasises his belief that tuberculosis in these situations is in a large proportion of cases local and often presents a tendency to spontaneous cure; even when abscesses and fistulæ have developed it is not uncommon to find the epididymis alone involved.

---

The incision advocated is made on the anterior aspect of the scrotum right through the tunica vaginalis to expose the testicle completely. If the testis, when split, appears healthy it is to be sutured at once with fine catgut, not chromicised. The separation of the epididymis is then begun at its tail, and all that is necessary is to incise the vaginalis both inwardly and outwardly in order to decorticate the organ from the testicle. The epididymis is drawn upwards and peeled off until it is retained only by a few small vessels, the vas deferens, and cellular tissue; this mass is ligatured and then cut. The vas deferens is cautiously snipped, care being taken not to injure the internal branch of the spermatic artery. The wound is drained for two days. Indications for castration are considered to be failure of epididymectomy to cure, and advanced coincident pulmonary lesions. In the acute type of tuberculosis also, Dr. Cumston favours castration, and regards conservative measures as out of the question; immediate and very radical removal is the only treatment which he recommends for such cases.

---

Bittorf contributes an elucidative article on Pericolitis to the *Mitteilungen aus den Grenzgebieten der Medizin und Chirurgie*, founded on a series of cases studied at Prof. von Strümpell's Clinic at Breslau. As early as 1853 Virchow had drawn attention to the frequency of old adhesive patches on the colon, chiefly at the flexures, which are to be met with in a fair percentage of bodies examined post mortem. It remained for Windscheid in 1889 to be the first to describe the clinical aspect of the condition, to which he gave the name Pericolitis. Bittorf argues that pericolitis is a well-marked clinical entity, easily recognisable at the bedside by those who have studied it. It is slightly more common in women than in men, affects all ages and conditions, and is usually acute in its onset. Fever is almost a constant sign, while constipation alternating with diarrhœa is almost the rule; in a few instances rigors have been observed. A leucocytosis may be present, as was found to be the case in the majority of patients examined by Bittorf, and the author lays stress on the increased excretion of indican in the urine. A very constant sign is localised tenderness over the colon, and it is important to differentiate this tenderness from the parietal tenderness in cases of peritonitis. In the latter superficial tenderness exists; in pericolitis deep pressure elicits the pain, which may also be accentuated by the passage of flatus within the lumen of the bowel at the affected spot. Rovsing's appendicular sign may be present, and where the pericolitis affects the ascending colon it may be difficult to differentiate the case from one of sub-acute appendicitis. In the latter and much more grave condition, vomiting and pain, irrespective of pressure, are the main features; the temperature is usually much higher, while the condition of the patient generally is much worse than in pericolitis. It is necessary to bear in mind, however, that the clinical picture of pericolitis may be very closely simulated by appendicitis, even where gangrene exists and the patients are in a very desperate condition. This is a point on which insufficient stress is laid in an otherwise exceptionally good article.

